# Insight into biological strategies and main challenges to control the phytopathogenic bacterium *Xylella fastidiosa*

**DOI:** 10.3389/fpls.2025.1608687

**Published:** 2025-06-27

**Authors:** Marwa Mourou, Giuseppe Incampo, Mariangela Carlucci, Davide Salamone, Stefania Pollastro, Francesco Faretra, Franco Nigro

**Affiliations:** Department of Soil, Plant and Food Sciences, University of Bari Aldo Moro, Bari, Italy

**Keywords:** *Olea europaea*, olive quick decline syndrome, xylem-bacterium, biological approaches, challenging aspects

## Abstract

*Xylella fastidiosa* is a xylem-restricted bacterium that can infect a wide range of host plants. The European Union classifies *Xylella fastidiosa* as a quarantine pathogen. Since its initial outbreak in the Apulia region of southern Italy in 2013, it has caused a severe disease in *Olea europaea*, known as olive quick decline syndrome (OQDS). Alarmingly, *X. fastidiosa* has continued to spread and establish itself in several European countries, including Spain, France, and Portugal. In response, researchers have conducted numerous studies to identify effective strategies for limiting the spread of the bacterium and its primary insect vector, *Philaenus* sp*umarius* L. These efforts have explored a wide range of approaches, including sustainable treatments, biological control agents, plant breeding techniques, and vector management strategies. This review aims to summarize the current research landscape and highlights the potential for improved management of this significant phytopathogen. The ongoing threat posed by *X. fastidiosa* underscores critical challenges for the future of agriculture in Europe and beyond. Furthermore, we identify key areas that remain poorly understood and require further investigation to develop effective and reliable control measures for this pathogen.

## 1 Introduction

*Xylella fastidiosa* is a Gram-negative bacterium characterized by its stringent growth requirements. It can infect a wide range of plant species up to 700 (European Food Safety Authority (EFSA) et al., 2023) either causing disease or persisting asymptomatically. The bacterium is non-motile and non-flagellated, with rod-shaped, strictly aerobic cells that have rounded or tapered ends and exhibit numerous irregular ridges or folds on the cell wall surface. Its optimal growth temperature ranges between 26–28°C (Wells et al., 1987). *X. fastidiosa* colonizes the xylem vessels of roots, stems, and leaves, obstructing the transport of water and mineral nutrients by forming bacterial aggregates known as biofilms. These biofilms develop in two main environments: within the host plant and in the foregut of the insect vector (Chatterjee et al., 2008). This dual habitat ensures that the pathogen is consistently protected within hosts, making it largely inaccessible to antimicrobial treatments.

Moreover, *X. fastidiosa* is capable of both upstream and downstream movement via twitching motility, which is mediated by type IV pili (Meng et al., 2005). Twitching motility refers to surface translocation across moist environments that does not rely on flagella. Specifically, pili of types I and IV are involved in twitching, biofilm formation, and cell-to-cell adhesion (Li et al., 2007).

Currently, the scientific community recognizes three main subspecies of *X. fastidiosa, fastidiosa*, *multiplex*, and *pauca* as the primary taxonomic divisions (Marcelletti and Scortichini, 2016). Additional subspecies, such as *sandyii*, *morus*, and *tashke*, have been proposed, and the taxonomy remains dynamic due to the continuous accumulation of genomic data and differentiation through multilocus sequence typing (MLST) (Maiden et al., 1998; Burbank and Ortega, 2018).

A particularly destructive subspecies, *pauca*, has evolved in Italy over the past decade generating an unprecedented plant health crisis, severely affecting olive trees. However, in United States, the *fastidiosa* subspecies has posed significant challenges to grapevines over the past 140 years. The timeline of *X. fastidiosa*’s detection and global spread underscores the pathogen’s complex biology and highlights the obstacles faced in diagnosis and disease management. It took 70 years to confirm that the pathogen is insect-transmitted (Winkler, 1949), nearly a century to determine that it is a bacterium rather than a virus (Goheen et al., 1973), and over 130 years to recognize its ability to spread via asymptomatic, infected plant material (Saponari et al., 2013). A recent economic analysis by the European Union estimated that, under a full-spread scenario, *X. fastidiosa* could cause annual damage of approximately €5.5 billion across Europe (European Commission, 2023). As Europe transitions toward more sustainable agriculture, the use of chemical agents previously employed to manage bacterial diseases is being restricted or banned (Boix-Fayos and de Vente, 2023). A recent comprehensive review (Wang et al., 2024) identified diseases caused by *X. fastidiosa* as among the most significant threats to plant health in the 21^st^ century.

Although the global outbreak of this pathogen has spurred considerable research into its biology and control, major knowledge gaps persist. The quest for an effective remedy remains a collective challenge that involves not only researchers and agricultural experts but also farmers and the general public. The urgency of the outbreaks and the associated economic burden underscore the need for a viable solution that will naturally garner widespread support among stakeholders.

In this review, we first assess the global economic impact of *X. fastidiosa*. We then compile key findings from recent studies on biocontrol and management practices, strategies to limit pathogen spread, and the implementation of phytosanitary measures. Finally, we highlight critical challenges that must be addressed in the future to achieve effective and sustainable disease control.

## 2 Economic and social impact

The insect-transmitted bacterium *X. fastidiosa* has a broad host range and poses significant economic and social threats to the agricultural and horticultural sectors. Major outbreaks have been recorded in Italy, Spain, France, and Portugal regions previously unaffected by this pathogen, which was historically restricted to the Americas.

The outbreak in Apulia, Italy, since 2013 has devastated more than 100 kilometers of olive-growing territory, affecting approximately 54,000 hectares and around 5 million olive trees, resulting in a 10% reduction in national olive oil production (White et al., 2020; Frem et al., 2021b). Estimated economic losses over the next 50 years range from €1.5 to €5.9 billion, accounting for reduced production as well as damage to landscapes and cultural heritage (D’Attoma et al., 2019; Schneider et al., 2020). Between 2016 and 2018 alone, production losses amounted to €390 million, while socio-ecological damages were estimated at €1,059 per hectare (Olivicola and Nazionale, 2019; Frem et al., 2021b).

In California, losses include $104 million annually due to yield reductions, management efforts, and regulatory measures (Tumber et al., 2014). The Pierce’s Disease Control Program, established to mitigate *X. fastidiosa*-related impacts, invested approximately $544 million between 1999 and 2010, mostly funded by federal sources (Hopkins and Purcell, 2002). Farmers in California bear $51.1 million annually in plant losses, with potential total costs reaching $185 million per year (Hopkins and Purcell, 2002; Tumber et al., 2014).

In Brazil, *X. fastidiosa* has caused Citrus Variegated Chlorosis (CVC), resulting in 120 million citrus plant infections in the 2000s and associated losses of approximately $110 million (Bové and Ayres, 2007; Gonçalves et al., 2011).

Across the European and Mediterranean regions, significant yield reductions have been observed: 78% in olives, 16.1% in citrus, 5.3% in grapes, and 0.6% in almonds. This translates into an estimated $12.44 billion in lost agricultural production (Cardone et al., 2022). The European Commission’s Joint Research Centre projects EU-wide costs could reach €5.5 billion annually, including €700 million in export losses.

In Lebanon, if infected grapevines are not replaced, revenue losses are projected to range from $11 million over four years to $82.44 million over 30 years (Frem et al., 2021a). In the Balearic Islands, almond orchard area declined from 29,789 ha in 2010 to 11,814 ha in 2019, primarily due to *fastidiosa* infection (Olmo et al., 2021). Additionally, *X. fastidiosa* subsp. *pauca* ST80 poses a significant threat to olive production, with symptoms resembling those caused by the aggressive Italian ST53 strain (Giampetruzzi et al., 2017). The California control program has demonstrated the importance of intergovernmental collaboration (e.g., USDA and state authorities) in mitigating economic losses (Tumber et al., 2014). In Europe, effective disease management is crucial, as subspecies *multiplex*, *pauca*, and *fastidiosa* continue to show expansion potential, driven more by human trade and vector dynamics than by climatic factors (Godefroid et al., 2019; Schneider et al., 2021). *X. fastidiosa* not only transforms landscapes but also endangers centuries-old agricultural traditions, particularly in olive-growing regions of Italy. Resistance to eradication and control measures has emerged, complicating disease management efforts (Martelli et al., 2016; White et al., 2020). Proactive prevention and sustained research are essential to curb the pathogen’s spread and mitigate its impact. Global projections for wine industry losses due to *X. fastidiosa* range from $2.3 to $7.9 billion over the next 50 years (Hafi et al., 2017). Indirect costs including job losses, reduced tourism, and trade restrictions further compound the pathogen’s socioeconomic impact (Schneider et al., 2021). Thus, *X. fastidiosa* exemplifies the urgent need for coordinated, cross-regional strategies to effectively confront one of the most pressing plant health crises of our time.

## 3 Measures adopted for *X. fastidiosa* biocontrol and management

*Xylella fastidiosa* is a fast-spreading bacterial pathogen that poses a serious threat to global agriculture, with no effective cure currently available. Ongoing efforts to manage this phytopathogen include the application of control measures on infected plants, government-regulated interventions, and the use of advanced molecular diagnostic tools.

These strategies include the adoption of resistant or tolerant plant cultivars, the implementation of stringent quarantine and eradication measures to remove infected plants, and the control of insect vectors -such as sharpshooters- through chemical or biological methods. Cultural practices, including pruning and sanitation, contribute to reducing bacterial reservoirs. In parallel, ongoing research is investigating the potential of beneficial microbes and natural antagonists as effective biological control agents. Collectively, these integrated approaches aim to achieve sustainable management of *X. fastidiosa* outbreaks and to protect vulnerable crops and ecosystems.

Indeed, these approaches can be broadly categorized into four main approaches: management of infected plants, deployment of resistant cultivars, application of antimicrobial substances to inhibit bacterial growth, and control of insect vectors. Importantly, numerous research studies have investigated diverse control strategies, including the use of natural compounds and microbial antagonists (**Table 1**).

**Table 1:** Summary of the main biological control measures worldwide applied against *X. fastidiosa*.

Biological/chemical measure	Host/Subspecies	Sequence Type/ strain	Methodology	Location	Reference
Antibiotics, olive mill wastewaters (OMV), phenolics, fungal extracts and toxins	Olive/*pauca*	Salento-1/ST53	Agar disk diffusion and agar disk dilution methods	Italy	(Bleve et al., 2018)
*Methylobacterium mesophilicum*	Sweet orange/*pauca*	-	*In vitro* broth dilution test using bacteria culture filtrate	Brazil	(Lacava et al., 2004, 2009)
Endophytic and epiphytic microorganisms	Olive/*pauca*	ST53	An *in vitro* dual culture method was employed to evaluate antagonistic activity, and well diffusion assays using culture filtrates were conducted to screen for antimicrobial activity.	Italy	(Mourou et al., 2022)
*Bacillus* spp.	Olive/*pauca*	ST53	The dual culture method on solid nutrient media was used to assess antagonistic interactions, while antimicrobial activity was evaluated through well diffusion assays using culture filtrates.	Italy	(Zicca et al., 2020)
Pomegranate peel extract	Olive/*pauca*	De Donno	The broth microdilution method was used to assess both planktonic growth and biofilm formation, while *in vivo* evaluations were conducted through endotherapeutic treatments in olive trees.	Italy	(Rongai et al., 2023)
Endophytes (*Pseudomonas* *fluorescens*, *Achromobacter xyloxosidans*, *Cochliobolus* sp.)	Grapevine *fastidiosa*	-	*In vitro* and *in planta* bioassays were performed, including assessment of symptom development and quantification of *Xylella fastidiosa* titer in planta using qPCR. Pathogen inoculation was carried out through vacuum infiltration and needle injection methods.	USA	(Rolshausen et al., 2018)
Nisin A	*Nicotianabenthamiana*/*pauca*	strain A0PT1	Viable-qPCR, spot assay, turbidity reduction assay, fluorescence microscopy, and transmission electron microscopy, RPLC-ESI-MS/MS analyses	Italy	(Sabri et al., 2024b)
*Paraburkholderia phytofirmans*	Grapevine/*fastidiosa*	Strain *Temecula*	The droplet puncture method was used to measure bacterial population size, disease severity, diffusible signal factor (DSF) production, and plant gene expression.	USA	(Lindow and Baccari, 2018; Baccari et al., 2019; Lindow et al., 2024)
Phage MATE 2	Olive/*pauca*	strain A0PT1	Genomic and electron microscopy analyses, spot assay, and broth dilution assay	Italy	(Sabri et al., 2024a)
*Curtobacterium flaccumfaciens*	*Catharanthus roseus/pauca*	–	Stem puncture inoculation with bacterial cultures was followed by evaluation of disease symptoms. PCR primers were designed for *Curtobacterium flaccumfaciens* to verify its presence in plant tissues and to complement an existing assay for *Xylella fastidiosa*.”	Brazil	(Lacava et al., 2007)
Bacteriophages	Grapevine/*fastidiosa*	Strain *Temecula 1*	Serial dilution spot assays were performed on overlays using a panel of 50 *Xylella fastidiosa* isolates as hosts. Grapevines were inoculated with both bacteria and phage to evaluate therapeutic and prophylactic phage treatments. Quantitative reverse transcription PCR (qRT-PCR) was used to detect phage and *X. fastidiosa*, and twitching motility was also assessed.	USA	(Das et al., 2015)
Transfer of bacteriophages by insects	Grapevine/f*astidiosa*	Strain Temecula	Rearing of glassy-winged sharpshooters (GWSS, *Homalodisca vitripennis*) was followed by feeding them on cowpea plants (*Vigna unguiculata* subsp. *unguiculata*) infected with the virulent phage Paz. Phage uptake by GWSS and its subsequent transmission to plants were assessed using qRT-PCR with specific primers targeting phage Paz.	USA	(Bhowmick et al., 2016)
*Leuconostoc mesenteroides* strain MS4-derived bacteriocins	Olive/*pauca*	ST53	Spot assays, fluorescence microscopy (FM), and transmission electron microscopy (TEM) were conducted, along with in planta assays involving stem inoculation using an insulin syringe on one-month-old *Nicotiana benthamiana* plants.	Italy	(Sabri et al., 2025)

### 3.1 Management of infected plants and implementation of phytosanitary measures

Currently, various initiatives are underway to establish regulations aimed at limiting the dissemination of all *Xylella fastidiosa* subspecies through the management of infected plants. In the European Union, quarantine restrictions are governed by Regulation (EU) No. 2016/2031 and supplemented by phytosanitary measures designed to prevent the introduction and spread of the pathogen across the region (European Parliament, and Council of the European Union, 2016). In Italy, regulations require the southeastern region to define a demarcated area, consisting of an infected zone and a surrounding buffer zone, to enhance disease management. The infected zone must include at least a 50-meter radius around infected plants, while the buffer zone ranges from 1 to 5 kilometres depending on the epidemiological context. In Portugal, following official confirmation of bacterial presence, containment and eradication measures must be promptly implemented under Implementing Regulation (EU) 2020/1201 (REGULATION (EU) 2020/1201) and Regulation (EU) No. 2016/2031. To ensure proper implementation and compliance, the national phytosanitary authority (DGAV), as mandated by Decree-Law No. 67/2020 (September 15), is responsible for delineating demarcated zones, establishing eradication protocols for the pathogen, and regulating the movement of cultivated plants within infected and buffer zones (Loureiro et al., 2023). Eradication of infected plants has occasionally proven effective in containing early-stage outbreaks, especially when the pathogen is detected only sporadically. Notable cases include the successful eradication of a limited infected area in Germany in March 2018, following its first detection in July 2016 (EPPO Global Database, n.d.), and the containment of *X. fastidiosa* in Southern California through a coordinated plan aimed at preventing the northward spread of Pierce’s disease (Bruening et al., 2014). However, the success of such eradication and containment strategies largely based on plant removal depends heavily on the cooperation of farmers, local communities, and political authorities. Where such cooperation is lacking, containment efforts are significantly undermined (Vicent and Blasco, 2017). Pruning of infected plants is another strategy employed to alleviate symptoms and attempt regeneration, especially since the bacterium typically migrates from terminal shoots commonly targeted by infected vectors toward the plant’s main stem. Pruning, alone or in combination with other treatments, has been documented as a mitigation strategy in several hosts, including oleander and citrus (Vandamme and Mortelmans, 2019), grapevine (Baccari et al., 2019), and almond (Sieiro et al., 2020). Another plant-targeted approach under exploration is “cold therapy,” based on the bacterium’s sensitivity to low temperatures (Feil and Purcell, 2001). Cold treatments have been proposed as a strategy to reduce pathogen loads in infected grapevines (Meyer and Kirkpatrick, 2008; Lieth et al., 2011); however, the applicability of this method to other plant species remains uncertain.

### 3.2 Plant breeding and the use of tolerant/resistant cultivars

An essential strategy for long-term management of *X. fastidiosa* involves the exploitation of resistant or tolerant cultivars, a method that has proven successful in managing other plant pathogens in the past (Krivanek and Walker, 2005; De Souza et al., 2009). Field studies in the Apulia region have shown that the widely cultivated olive varieties Ogliarola salentina and Cellina di Nardò are highly susceptible to *X. fastidiosa*, while resistance traits have been identified in the cultivars Leccino and FS17 (D’Attoma et al., 2019). For instance, Montilon et al., 2022 demonstrated that Ogliarola salentina and Cellina di Nardò exhibited greater sensitivity to infection than Leccino, which was linked to more frequent vessel occlusions, including tyloses, gums, and pectin deposition (Montilon et al., 2022). Cardinale et al., 2018 reported significantly lower bacterial cell concentrations in the stems of Ogliarola Salentina (Cardinale et al., 2018). Other studies have revealed that Leccino trees harbour bacterial populations up to 100 times lower than those in susceptible cultivars and can withstand *X. fastidiosa* infections under greenhouse conditions (Giampetruzzi et al., 2016; D’Attoma et al., 2020). Moreover, electron microscopy analyses confirmed that Leccino olives exhibited greater resistance to symptom development compared to Cellina di Nardò (Surano et al., 2022). Symptom severity of olive quick decline syndrome (OQDS) varies notably among different olive genotypes. In susceptible cultivars, such as Cellina di Nardò and Ogliarola Salentina, lower levels of tylosis induction may facilitate bacterial movement through xylem vessels (Petit et al., 2021). Indeed, higher levels of xylem vessel occlusion have been documented in these susceptible varieties relative to Leccino (De Benedictis et al., 2017). Further evidence comes from Mauricio et al (Mauricio et al., 2019), who evaluated field resistance to *X. fastidiosa* in 264 hybrids derived from Citrus reticulata × Citrus sinensis and pear orange. Healthy plants were grafted with infected material. The results showed that hybrid progenies remained asymptomatic and harbored negligible bacterial loads, whereas all pear orange plants displayed symptoms of citrus variegated chlorosis (CVC) and tested positive for infection. In grapevines, resistant varieties exhibit approximately 20% xylem occlusion, in contrast to up to 60% occlusion in susceptible cultivars. A comprehensive evaluation of 72 plant species has identified varying levels of tolerance or resistance, with Vitis, Citrus, and Prunus species being among the most extensively studied groups (Sun et al., 2013; Authority et al., 2023).

### 3.3 Biocontrol agents, microbial and plant extracts involved in bacterial growth inhibition

#### 3.3.1 Biocontrol agents

Several products, including antibiotics, metal and mineral compounds, natural substances, microbial agents, plant extracts, and antibacterial compounds, have been evaluated in both *in vitro* and field studies against *Xylella fastidiosa*. Research on biological agents has primarily focused on the endophyte *Paraburkholderia phytofirmans*, avirulent *X. fastidiosa* strains (notably EB92-1 and DPD1311), and lytic bacteriophages (Zhang et al., 2015; Saponari et al., 2019; Sabri et al., 2024a). Moreover, other studies have highlighted the antagonistic and antibacterial properties of epiphytic and endophytic bacteria against *X. fastidiosa* (Mourou et al., 2022). Furthermore, it has been demonstrated that specific endophytic microorganisms can reduce the virulence of *X. fastidiosa*, either by competing with the pathogen for ecological niches or by producing compounds that interfere with its pathogenicity (Azevedo et al., 2016) (Dourado et al., 2015). Similarly, Baccari et al., 2019 evaluated the efficacy of endophytes introduced into grapevines through stem punctures (Baccari et al., 2019). This approach led to a notable reduction in disease severity, suggesting that these biological agents can mitigate infection by stimulating the plant’s resistance responses. The tested strain showed significant effectiveness in managing Pierce’s disease and could be conveniently applied via foliar spraying as a viable control strategy (Baccari et al., 2019). Microorganisms such as *Curtobacterium flaccumfaciens* and *Methylobacterium mesophilicum* in citrus, various fungal species in grapevines, and *Paraburkholderia phytofirmans* recently identified in olives exhibit the ability to suppress bacterial growth through competition or by producing metabolites that modulate bacterial virulence (Lacava et al., 2004, 2009; Baccari et al., 2019; Lindow et al., 2024). To date, no significant differences in the mitigation of Olive Quick Decline Syndrome (OQDS) symptoms or in the reduction of new infections have been observed following preventive applications of *P. phytofirmans* in the Italian ‘De Donno’ olive pathosystem. Recent studies have investigated the role of microbial endophytes in the sapwood of Apulian olives as a potential solution for managing *X. fastidiosa*, with evidence supporting their contribution to resistance traits in several olive cultivars (Giampetruzzi et al., 2020; Hanani et al., 2021; Vergine et al., 2024). Notably, the composition of plant-associated microbial communities significantly influences disease susceptibility (Mitter et al., 2019; Compant et al., 2021). The growing interest in using microbial endophytes as biocontrol agents against phytopathogens (Mitter et al., 2019; Morelli et al., 2020), along with promising results in the control of strain Temecula1 (Kirkpatrick et al., 2003; Rolshausen et al., 2018), has spurred the search for similar strategies to combat *X. fastidiosa* strain De Donno and its associated disease impacts. In particular, Vergine et al., 2024 reported a marked dysbiosis caused by *X. fastidiosa* in the susceptible cultivar ‘Cellina di Nardò’, which was absent in the more resistant ‘Leccino’ cultivar that maintained greater microbial diversity (Vergine et al., 2024). The tendency of endophytes to be displaced by *X. fastidiosa* during the course of infection was further confirmed by (Giampetruzzi et al., 2020), who found this phenomenon to be more pronounced in the susceptible cultivar ‘Kalamata’ compared to the resistant ‘FS-17^®^’. Additionally, strains of *Methylobacterium mesophilicum* and *M. radiotolerans* have been noted for their ability to secrete siderophores iron-chelating (Fe³^+^-binding) compounds that can enhance the efficacy of biocontrol agents by improving their competitive advantage within the plant microbiome (Lacava et al., 2008).

In summary, microbial agents represent a promising and environmentally sustainable strategy for the management of *Xylella fastidiosa*. However, their efficacy remains inconsistent across different host plant species and environmental conditions. Current challenges include variable performance under field conditions, largely due to the complex interactions among host plants, microbial communities, and the pathogen itself, as well as the need for optimized application methods.

Continued research is essential to improve the effectiveness of these biocontrol strategies and to facilitate their integration into comprehensive, multifactorial disease management programs.

#### 3.3.2 Microbial and plant extracts

Interestingly, numerous plant-derived natural compounds have been extensively studied for their potential activity against *X. fastidiosa*. In this context, (Maddox et al., 2010; Aldrich et al., 2015) demonstrated the *in vitro* inhibitory effects of several substances on the bacterium, including polyphenols, azadirachtin A, hesperidin, and radicinin. Additionally, (Bleve et al., 2018) examined the antimicrobial properties of various plant-based phenolic compounds such as 4-methylcatechol, catechol, and oleuropein as well as filtered fractions of olive mill wastewater (OMW), *Trichoderma* spp. culture extracts, and fungal toxins, evaluating their applicability as natural antimicrobial agents. All phenolic compounds tested showed inhibitory activity against *X. fastidiosa* strain De Donno, although this effect was generally bacteriostatic and reversible. For example, ophiobolin A and gliotoxin exhibited bacteriostatic effects, whereas a crude extract derived from *T. citrinoviridae* demonstrated bactericidal properties. Notably, the addition of microfiltered OMW fractions to the culture medium significantly influenced the growth of the De Donno strain. Similarly, other classes of natural compounds such as coumarins, stilbenes, and flavonoids have been tested *in vitro* against *X. fastidiosa* strains associated with Pierce’s disease. Collectively, these compounds were found to inhibit bacterial growth effectively, as reflected by their low minimum inhibitory concentrations. Furthermore, the structural diversity among phenolic compounds contributed to varied levels of antagonistic activity. Among the most potent inhibitors were catechol, caffeic acid, and resveratrol (Maddox et al., 2010). Comparable assays evaluated the *in vitro* effects of additional phenolic compounds, including gallic acid, epicatechin, and resveratrol, on the growth of *X. fastidiosa*. While none of these substances completely suppressed bacterial proliferation, some such as epicatechin and gallic acid significantly reduced cell surface adherence. Moreover, resveratrol treatment was associated with a decrease in cell-to-cell aggregation (Lee et al., 2020). Studies on essential oils (EOs) have demonstrated their potential utility in managing *X. fastidiosa*, as their antimicrobial effectiveness against a range of phytopathogens and pests has been validated through numerous *in vitro* studies (Bajpai et al., 2010; Santiago et al., 2018; Raveau et al., 2020). Santiago et al., 2018 investigated the antibacterial effects of sandalwood and patchouli essential oils, reporting promising outcomes; both oils exhibited notable antimicrobial activity, highlighting their potential as biological sources for the development of novel plant protection products (Santiago et al., 2018). Montesinos et al. assessed the efficacy of eucalyptus essential oil against 11 phytopathogenic bacterial species from six distinct genera (Montesinos et al., 2023). The study showed that all tested pathogens were susceptible to the oil, with *X. fastidiosa* and *Xanthomonas fragariae* being particularly affected. The oil exerted a strong bactericidal effect, including lytic activity against three *X. fastidiosa* subspecies examined in the study (Montesinos et al., 2023). Additionally, efforts have been made to mitigate *X. fastidiosa* infections and associated symptoms by targeting the pathogen’s diffusible signal factors (DSFs), which play a key role in cell–cell communication and virulence. These DSFs have been explored for their potential use in the biological control of *X. fastidiosa*-associated diseases in both grapevine and citrus plants (Lindow et al., 2014; Caserta et al., 2017). The application of the plant growth regulator abscisic acid (ABA) as a foliar spray on infected ‘Pinot Noir’ and ‘Cabernet Sauvignon’ grapevines has also been investigated. In treated ‘Pinot Noir’ vines, ABA application led to a marked increase in the production of phenolic compounds in xylem sap, along with an overall improvement in plant health compared to untreated controls. These findings indicate a favorable correlation between ABA treatment and enhanced phenolic content, suggesting an indirect antibacterial effect of the hormone (Meyer and Kirkpatrick, 2011). Other innovative approaches for controlling *X. fastidiosa* include the use of fosetyl-aluminum nanocrystals encapsulated in chitosan (Baldassarre et al., 2020) and antimicrobial peptides (AMPs) (Baró et al., 2020). Despite their potential, these mineral- and peptide-based strategies have not yet achieved consistent success in disease suppression, indicating the need for further development and optimization.

**Figure 1:**
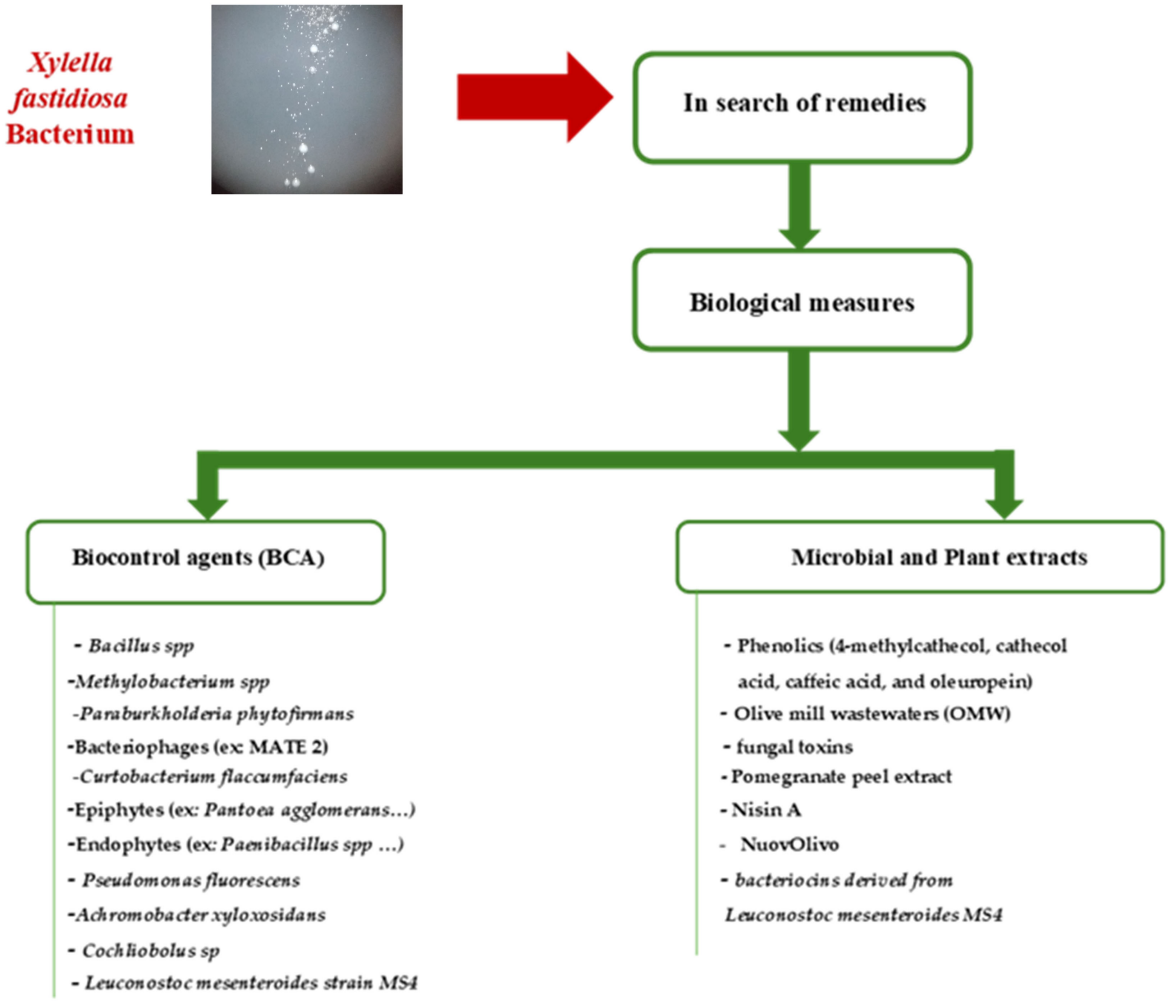
Main biological measures involved in *Xylella fastidiosa* control: (Biocontrol agents, microbial and plant extracts).

Among AMPs, cecropin B (CB) has shown bactericidal activity against a broad spectrum of plant-pathogenic bacteria, including members of the genera *Erwinia*, *Xanthomonas*, *Pseudomonas*, and *Clavibacter* (Ishida et al., 2004). Transgenic grapevines engineered to express CB exhibited only mild symptoms following inoculation with *X. fastidiosa*, and the bacterial invasion was slow and limited. A significant reduction in both bacterial proliferation and colony size was observed, likely due to reduced CB production within the host (Ishida et al., 2004). Overall, the successful control of *X. fastidiosa* typically involves the synergistic action of multiple antimicrobial agents, as summarized in **Figure 1**.

Plant-derived compounds, such as essential oils and antimicrobial peptides, represent promising tools for the development of more sustainable control strategies against *X. fastidiosa*. These substances may contribute to enhancing host immune responses and disrupting key bacterial physiological processes. Despite their potential, several challenges limit their practical application. These include variable efficacy under field conditions, limited chemical stability, potential phytotoxic effects, and difficulties in achieving uniform and effective application. Future research should prioritize the elucidation of their mechanisms of action and evaluate synergistic formulations that combine agents with direct antimicrobial activity and those capable of activating plant defense pathways, including the expression of resistance-related genes.

## 4 Control of insect vectors: entomopathogenic and predatory organisms

The absence of curative treatments for plants infected by *Xylella fastidiosa* makes vector management the primary strategy for mitigating the spread of the pathogen in affected areas. One promising strategy to limit the spread of *X. fastidiosa* involves targeting its insect vectors, particularly *Philaenus* sp*umarius*, which is responsible for transmitting the pathogen to a wide range of host plants. Due to the unique characteristics of its transmission persistent, non-circulative, and lacking a latency period *X. fastidiosa* is difficult to disrupt once acquired by its insect vectors. Therefore, vector control efforts aim to reduce transmission by lowering or eliminating vector populations, particularly those that visit susceptible host plants.

Biological control agents (BCAs), especially those based on predatory insects and entomopathogenic organisms, have emerged as effective tools for vector management. Studies have demonstrated that the predatory insect *Zelus renardii* can significantly reduce *P.* sp*umarius* populations and, consequently, lower the incidence of *X. fastidiosa* infections in olive trees, supporting the use of natural predators as biocontrol agents (Liccardo et al., 2020). The biocontrol model proposed by Liccardo et al. (2020) is based on both laboratory and field trials, highlighting the effectiveness of a dual approach that combines predation and inundation techniques to mitigate the threat posed by *X. fastidiosa*. However, the introduction of predatory insects also raises ecological concerns, including the risk of unintended environmental consequences such as uncontrolled proliferation of the biocontrol agent or disruption of existing ecological balances within olive groves.

In a similar vein, another study emphasized the value of integrating chemical and physical control methods with biological interventions to manage vector populations more effectively (Picciotti et al., 2021).

Beyond predatory insects, entomopathogenic nematodes (EPNs) and fungi have also gained prominence as potential BCAs targeting the *X. fastidiosa* vector. El-Khoury et al., 2024 evaluated the pathogenicity of different EPN and fungal strains, demonstrating the efficacy of species such as *Beauveria bassiana* and *Lecanicillium muscarium* against *P.* sp*umarius* (El-Khoury et al., 2024).

Numerous measures to reduce nymphal populations have been tested through experimental trials in Italy and Spain. Dongiovanni et al., 2018 conducted a three-year study comparing the efficacy of different foliar sprays targeting weed and ground vegetation management as a means to reduce juvenile populations of *Philaenus* sp*umarius* and *Neophilaenus campestris* (Dongiovanni et al., 2018b). The authors also demonstrated that the application of orange oil significantly reduced nymphal populations, suggesting its effectiveness in managing early vector stages (Dongiovanni et al., 2018b). However, its efficacy appears limited to nymphs inhabiting herbaceous ground cover and may be less practical in environments where undergrowth control is difficult or once vectors reach adulthood.

In Portugal, vector control strategies include the use of plant protection products that comply with safety standards for human health and the environment. Recently, certain plant protection products have been granted exceptional authorization for use in vector management. Dongiovanni et al., 2018 reported that acetamiprid a neonicotinoid insecticide exhibited substantial toxicity against *P.* sp*umarius*. Nevertheless, the overuse of contact insecticides can accelerate resistance development in pest populations and negatively impact beneficial arthropods (Dongiovanni et al., 2018a).

In another study, Dongiovanni et al. (2017) showed that citrus oil was effective against nymphs when applied at high volumes (2,000 L/ha), although its efficacy was confined to immature stages. Despite its toxicity, some publications argue that acetamiprid does not significantly interfere with bacterial inoculation, as vectors exposed to this insecticide showed lower susceptibility compared to those treated with other insecticides (Lago et al., 2022). Similarly, Bethke et al., 2001 documented the effectiveness of a neonicotinoid in reducing vector populations in California (Bethke et al., 2001).

Behavioural studies by (di Domenico et al., 2019) found that male and female *P.* sp*umarius* responded differently to varying doses of citrus oil, demonstrating either at-traction or repulsion. Additionally, Lago et al., 2022 evaluated the protective effects of kaolin, a clay-based particle film, which acts as a mechanical barrier against insect vectors such as *Homalodisca vitripennis*, a known *X. fastidiosa* vector. Kaolin was shown to deter insect feeding and oviposition, ultimately leading to vector mortality (Lago et al., 2022).

More recently, interest in natural enemies of spittlebugs has grown in Europe. For instance, Reis et al. (Reis et al., 2018) reported the identification of egg parasitoids in Portugal, while Mesmin et al. (Mesmin et al., 2020) documented the presence of the egg parasitoid Ooctonus vulgatus Haliday in Corsica.

Additionally, natural predation by birds and small reptiles targets both nymph and adult stages of Cicadellinae, while larvae of coccinellids and lacewings have been observed feeding on egg masses. These interactions support the development of sustainable integrated pest management (IPM) programs. Effective IPM will require precise timing of interventions, careful evaluation of novel formulations, and the optimization of treatment volumes and methods (Liccardo et al., 2020).

In France, Grandgirard et al., 2008 tested the release of natural enemies such as *Gonatocerus* spp., egg parasitoids of sharpshooter vectors. Their study reported a 95% reduction in vector populations within seven months of release (Grandgirard et al., 2008, 2009). In a complementary approach, other research explored the isolation of insect-specific viruses capable of reducing bacterial adhesion to vectors, indicating their potential as biopesticides (Stenger et al., 2009).

On the whole, these findings underscore a key challenge in biological control: ensuring the specificity and effectiveness of BCAs in targeting the appropriate insect vectors. In this context, microbial pathogens represent an environmentally friendly alternative to chemical insecticides and help reduce the ecological footprint of agricultural practices. The successful development and implementation of BCAs also require a deep understanding of *X. fastidiosa* ecology and genetics, particularly the genomic diversity among its subspecies. Research by Vanhove et al. (2019) revealed strain-specific traits that influence host range and vector interactions, emphasizing the importance of genomic analysis in optimizing biocontrol strategies.

These potential differences in strain susceptibility have significant implications for biocontrol program design, as variations in response to entomopathogenic agents could influence overall effectiveness. Additionally, *X. fastidiosa* possesses a remarkable capacity for horizontal gene transfer (HGT), further complicating its evolutionary trajectory. The acquisition of genetic material through conjugation may enhance virulence and promote adaptation to control strategies, thereby highlighting the need for continuous monitoring and adaptive management systems (Burbank and Van Horn, 2017). Understanding these evolutionary and ecological mechanisms enables researchers to select appropriate BCAs and incorporate them into comprehensive, integrated disease management frameworks. In this regard, Kyrkou et al. (2018) provide an overview of various control strategies, emphasizing the incorporation of BCAs into integrated pest management (IPM) systems that aim to balance crop protection with ecological sustainability and economic viability.

### 4.1 Challenges to understand and control *X. fastidiosa*: critical points and future perspectives

In recent years, several strategies have been explored to mitigate the impact of *X. fastidiosa*, including the use of natural plant extracts such as those derived from olive leaves (Vizzarri et al., 2023). These extracts have demonstrated *in vitro* antibacterial activity against various phytopathogens, including *X. fastidiosa*. Although biodegradable and environmentally sustainable, their practical application *in vivo* presents several challenges. These include the need to identify optimal dosages which can vary depending on plant species, age, and phenological stage ensuring effective translocation of the active compounds within the xylem and developing appropriate treatment protocols. Furthermore, repeated applications may be necessary, substantially increasing costs and thereby limiting the feasibility of this method as a large-scale disease management strategy. Another promising approach involves the use of microbial antagonists, such as *Leuconostoc mesenteroides* MS4. Culture filtrates from this strain have shown significant antibacterial activity, largely due to the production of bacteriocins that effectively inhibit *X. fastidiosa* growth (Sabri et al., 2025). These bacteriocins could potentially be applied in the field via trunk injection (endotherapy) to facilitate systemic distribution through the plant’s vascular tissues. However, endotherapy presents several limitations. Direct injection can damage the vascular system, potentially causing permanent internal lesions. Moreover, the systemic distribution of the compounds may be uneven and is often impeded by xylem occlusion, particularly in advanced stages of infection. An innovative and highly specific biocontrol strategy involves the use of bacteriophages. For example, the phage Mate 2 has shown lytic activity against *X. fastidiosa* and offers targeted suppression of the pathogen (Sabri et al., 2024a). Despite its promise, this approach also faces limitations. The high costs associated with research, development, and large-scale production of bacteriophage formulations may restrict widespread adoption. Additionally, bacteriophage-based treatments may offer only temporary relief, as the pathogen could mutate and develop resistance over time, thereby reducing long-term efficacy. A critical factor in the epidemiology of *X. fastidiosa* is the role of insect vectors, which are essential for pathogen transmission between plants. Any xylem-feeding insect has the potential to act as a vector, transmitting the bacterium without a latent period (Purcell and Finlay, 1979). Transmission efficiency is particularly concerning because *X. fastidiosa* colonizes the insect’s foregut, and even a minimal bacterial load is sufficient for inoculation (Hill and Purcell, 1995). Although transmission efficiency varies among insect species (Almeida et al., 2005), the mobility and feeding behavior of some vectors, especially their tendency to visit multiple plant species significantly increase the risk of widespread dissemination. Host plants that are irrigated during summer, such as grapevines in California (Almeida et al., 2005) or olives in southern Italy, as well as ornamental and riparian vegetation, provide ideal conditions for vector proliferation. These factors collectively make halting insect-mediated transmission of *X. fastidiosa* extremely challenging. Additionally, several challenges arise in the detection of *X. fastidiosa*. The bacterium can persist asymptomatically for extended periods such as in olive trees complicating the identification of infected plants based solely on visible symptoms. In this context, Ahmed et al., 2023 reported the use of a spectranomic approach for the early detection of asymptomatic infections in olives (Ahmed et al., 2023). However, the uneven distribution of the bacterium within plant tissues, along with latent periods that delay symptom expression, hinders the efficient selection of sampling sites for accurate infection assessment. Difficulties in controlling *X. fastidiosa* are further compounded by the characteristics of its host plants, many of which are woody species of historical and economic importance. The main challenges include landscape degradation, high costs associated with removing infected mature trees, prolonged recovery periods before replanting becomes productive, and the technical complexity of sampling large canopies with sufficient accuracy to detect the pathogen. Although approximately 700 plant species are currently recognized as potential hosts of *X. fastidiosa* (European Food Safety Authority (EFSA) et al., 2023), only a small fraction of these pathosystems have been studied in depth beyond initial disease reports. Grapevines, citrus, and olives are among the most thoroughly investigated. In contrast, limited data exist for almonds although research on Prunus species has recently increased as well as for blueberries, peaches, plums, coffee, and several other economically important crops. Notably, detailed information remains scarce for roughly 98% of known or suspected host species. Future research should prioritize the development of sustainable control strategies that exploit both naturally occurring microorganisms inhabiting the xylem niche and bioactive compounds of natural origin. This effort requires comprehensive metagenomic studies to identify novel microorganisms, assess their antimicrobial potential, and evaluate their capacity to persist within the xylem environment. Furthermore, advanced analytical techniques such as Gas Chromatography–Mass Spectrometry (GC-MS) and Nuclear Magnetic Resonance (NMR) spectroscopy should be employed to isolate and characterize bioactive molecules with inhibitory effects against *X. fastidiosa*. Equally important is the development of environmentally sustainable methods for introducing these microorganisms or compounds into the ecological niche occupied by *X. fastidiosa*. Ideally, an effective microbial antagonist or bioactive substance should be capable of entering the plant via the root system and reaching the xylem vessels, where the bacterium resides. The identification and selection of resistant olive cultivars also remain critical for the long-term management of the disease. However, reliance on a limited number of resistant varieties poses significant challenges, including reduced genetic diversity, the risks of monoculture landscapes, and the potential for pathogen adaptation or mutation that may overcome existing resistance mechanisms. To address these issues, it is imperative to expand the genetic diversity of cultivated varieties and implement innovative, integrated management strategies. The convergence of omics technologies with advanced analytical methods such as GC-MS and NMR spectroscopy will be pivotal in developing more effective, durable, and ecologically sound approaches to managing *X. fastidiosa*.

## 5 Conclusion

This review summarizes the principal strategies and ongoing challenges associated with the control of *X. fastidiosa*, a highly destructive and globally significant plant pathogen. For more than a century, the diseases caused by *X. fastidiosa* including Pierce’s disease in grapevines, Citrus Variegated Chlorosis (CVC), and more recently Olive Quick Decline Syndrome (OQDS) have threatened major agricultural systems. Despite decades of research, no curative solution has yet been found, and effective, long-lasting management strategies remain limited across the wide range of host species and diverse environmental contexts in which the bacterium thrives. Innovative and interdisciplinary approaches are urgently required to overcome the complex biology of *X. fastidiosa*, including its broad host range, efficient insect vector transmission, and ability to persist asymptomatically in reservoirs. A key component of progress will involve expanding research efforts beyond the best-studied pathosystems (e.g., grapevine, citrus, olive) to include under-researched hosts, such as almond, blueberry, coffee, and numerous ornamental or wild species that may act as silent reservoirs. The formulation of integrated disease management strategies must incorporate short-term, rapid-response measures such as vector suppression and phytosanitary containment with longer-term goals like breeding for resistance, endotherapy using microbial antagonists or bioactive compounds, and the development of environmentally sustainable biological control tools. These efforts should be supported by advanced technologies, including genomics, metagenomics, metabolomics, and precision agriculture platforms. Achieving tangible progress in the management of *X. fastidiosa* also requires robust collaboration among multidisciplinary research teams, policymakers, agricultural stakeholders, and growers. Effective knowledge transfer, shared surveillance data, and harmonized phytosanitary regulations across regions are vital for mitigating the spread and impact of this pathogen. Stakeholder engagement, particularly with farmers and local communities, is crucial to ensure the acceptance and practical implementation of control strategies. In conclusion, addressing the global threat posed by *X. fastidiosa* demands both scientific innovation and coordinated action. Through sustained collaboration and investment in research, it is possible to develop holistic, adaptive, and resilient solutions to safeguard plant health, agricultural productivity, and biodiversity in the face of this evolving pathogen.
